# The National Medical Union Conference

**Published:** 1913-10-25

**Authors:** 


### THE NATIONAL MEDICAL UNION CONFERENCE.

The decision of the National Medical Union, announced in the press last week, to convene a conference in London, may be expected to provide an additional stimulus to the non-panel movement. The coming conference will consist of delegates from all non-panel bodies, and its object will be to consider the question of the federation of all such associations throughout the country.

When the true numerical strength of the non-panel men is known, and their various associations have been linked up under a strong federating committee, there is no doubt that these men will represent a force to be reckoned with in any fresh legislation affecting medical affairs. Any non-panel man having no local association available could not do better than become a member of the National Medical Union (5 John Dalton Street, Manchester), which is the oldest and largest non-panel body, and which has been consistent in its policy throughout. Attention may also be directed to a letter appearing in the *Lancet* of October 18, from Mr. E. Arthur Dorrell, of 45 Welbeck Street, W., honorary secretary of the London Medical Committee, which is engaged in promoting the formation of non-panel associations in the County of London. Mr. Dorrell offers to give any information in his power, and in the course of his letter he says: "It is of extreme importance that non-panel men should keep actively in touch with each other and prepare by combination to meet the increasing attacks upon their interests. The British Medical Association has shown that the non-panel man can expect little assistance from the Association, and it is only through a body formed exclusively of non-panel men that he can obtain prominence for his views and any chance of being heard. It therefore behoves every man who has no intention of working under the panel system to become a member of the non-panel association in his division, and if such an association has not yet been formed in his division to make endeavours to start one."

As the practical results of the working of the Insurance Act appear, it is becoming clear that the lot of the panel man is by no means a uniformly happy one. No doubt in some cases a substantial rise in income has resulted, and here the quarterly cheque is the compensating factor; but there are some serious drawbacks which as time goes on will certainly result in the panel man becoming less satisfied with his position. It appears, for example, according to a recent decision, that the panel doctor is under a legal obligation to attend when sent for, and may be successfully sued for fees incurred should he refuse. A non-panel man is, of course, under no sort of legal compulsion to attend a patient. If the decision quoted is upheld on appeal the result will be that an insured person may proceed against his panel doctor both in the Law Courts and also under the Insurance Act Regulations. In the case referred to the Medical Service Sub-Committee had already dealt with the matter and had recommended that no action should be taken. Two opposite decisions were thus arrived at, and if medical men are to be harassed both by investigations by Insurance Committees and by actions at common law, we shall no doubt see the panel man seizing any opportunity to escape from the clutch of officialdom and legal compulsion by rejoining the ranks of the non-panellites, where he will be free from these irksome restrictions.

Returning for a moment to the pecuniary aspect of the matter, it was decided in some districts that to avoid some of the objectionable features of contract practice, payment for work done should be substituted for a capitation fee. The result in Manchester, where this system was adopted, is that for the first half of this year for every 7s. earned only 4s. is available, and, as the Report of the Medical Committee says, practitioners are "faced with the alternatives of reducing the work done or definitely accepting the rôle of philanthropists by order of the Government."

These considerations serve to illustrate some of the drawbacks attending medical service under the Insurance Act, and to justify the expectation of success in the efforts now being made in the non-panel cause.

